# Validation of the Mild Behavioral Impairment Scale (MBI-S) for brief self-assessment of Mild Behavioral Impairment in people without dementia

**DOI:** 10.1186/s12991-025-00566-w

**Published:** 2025-05-29

**Authors:** Paula Hinkl, Elmar Graessel, Nicolas Rohleder, Peter Landendoerfer, Thomas Kuehlein, Natascha Lauer, Anna Pendergrass

**Affiliations:** 1 Center for Health Services Research in Medicine, Department of Psychiatry and Psychotherapy, Uniklinikum Erlangen, Friedrich-Alexander-Universität Erlangen- Nürnberg (FAU), Schwabachanlage 6, 91054 Erlangen, Germany; 2 Chair of Health Psychology, Department of Psychology, Friedrich-Alexander-Universität Erlangen-Nürnberg (FAU), Erlangen, Germany; 3 Institute of General Practice and Health Services Research, Technical University of Munich, Munich, Germany; 4 Institute of General Practice, Uniklinikum Erlangen, Friedrich-Alexander-Universität Erlangen-Nürnberg (FAU), Erlangen, Germany

**Keywords:** Mild behavioral impairment, Neuropsychiatric symptoms, Checklist, Validation, Cross-sectional study

## Abstract

**Background:**

Mild Cognitive Impairment (MCI) and Mild Behavioral Impairment (MBI) are important constructs in the context of cognitive decline. MBI can be assessed with the Mild Behavioral Impairment Checklist (MBI-C). However, the instrument has deficits in psychometrics and content, thus indicating a need for improvement. The aim of this study was to develop a complementary short instrument, the Mild Behavioral Impairment Scale (MBI-S), designed to measure MBI as a short-term modifiable state criterion, and to validate it in a non-clinical sample of people 18 years of age or older.

**Methods:**

Most of the items on the MBI-S stem from the MBI-C and were chosen to represent the dimensions of the Neuropsychiatric Inventory Questionnaire. The MBI-S was validated on self-reported data from 175 individuals. In an item analysis, the discriminatory power and item difficulties were examined. Cronbach’s alpha was calculated to assess the internal consistency, and a principal component analysis was conducted to determine the structure of the instrument. Construct validity was established by testing four hypotheses about relationships between the MBI-S and other instruments by calculating correlation coefficients.

**Results:**

After the item analysis, two items were removed from the final version of the scale on the basis of insufficient discriminatory power and the finding that the internal consistency of the total score increased when the items were deleted. The principal component analysis yielded a single-component structure for the MBI-S. Two more items were excluded from the scale due to insufficiently low loadings on the extracted component. Cronbach’s alpha for the final eight-item scale was 0.79. The final MBI-S score was strongly related to that of the MBI-C and a loneliness score as well as moderately related to maladaptive coping. There was no association with respondents’ level of education.

**Conclusion:**

The MBI-S is a valid short instrument for the assessment of MBI. It has high test economy and measures current neuropsychiatric symptoms and their intensity as a state criterion. Therefore, the MBI-S can be used for the longitudinal measurement of MBI.

**Supplementary Information:**

The online version contains supplementary material available at 10.1186/s12991-025-00566-w.

## Background

Today, more than 55 million people worldwide are affected by dementia, which is now one of the ten leading causes of death worldwide [1]. It is caused by Alzheimer’s disease in around 60–70% of cases. As this neurodegenerative disease cannot yet be cured or reliably prevented, early detection in pre-dementia phases is important, as intervention can at least delay the development of dementia [2]. Cognitive impairment, especially when objectively measurable, often precedes the clinical stages of dementia and is considered an at-risk state referred to as Mild Cognitive Impairment (MCI), with conversion rates to dementia of 10–15% per year [3–5]. Since not all individuals with measurable cognitive impairment develop dementia, and some individuals convert more quickly than others, the identification of other non-cognitive factors that may influence progression to dementia and improve early detection is of great interest [6, 7]. Focusing on the neurobehavioral axis of change in dementia, research has shown that while neuropsychiatric symptoms (NPS) are common in manifest dementia [8], beginning behavioral and psychological changes already occur in preclinical stages and can be of predictive value [9, 10]. These NPS include disorders of mood, perception, or behavior and may for example appear as depressive symptoms, hallucinations, agitation, or sleep disturbances [11, 12]. Research has shown that they also occur in cognitively normal older adults, and they precede cognitive impairment and dementia [13, 14] while being associated with measurable cognitive decline [15]. In conclusion, research has suggested that NPS could be used to identify people with an increased risk of developing dementia in an early stage, independent of cognitive impairment. Such an identification can be implemented with the concept of Mild Behavioral Impairment (MBI), which represents a non-cognitive risk condition for the development of dementia [11].

According to the latest definition, MBI refers to persisting behavior or personality changes after the age of 50 years, including decreased motivation, affective dysregulation, impulse dyscontrol, social inappropriateness, or abnormal perception or thought content. It causes impairment in interpersonal, social, and work-related aspects of life [11]. In meta-analyses, the pooled prevalence of MBI in older people was reported to be 45.5% in MCI subjects and 17% in cognitively unimpaired participants [16]. MBI has been shown to increase the risk of developing dementia [17–19]. Further investigations of MBI are necessary to define its relationship with dementia and explore possible interventions, thus creating the need for reliable and valid instruments for measuring the construct.

Therefore, the Mild Behavioral Impairment Checklist (MBI-C) was developed as an instrument for assessing MBI on the basis of the criteria revised by the International Society to Advance Alzheimer’s Research and Treatment – Alzheimer’s Association (ISTAART-AA; [11, 20]). It consists of 34 items that are ideally answered by close caregivers but also by medical staff or by self-report. The instrument is freely available (https://mbitest.org/). It has been translated into several languages, including German [21]. For the English version, exploratory factor analyses with both proxy-reported and self-reported data from non-demented individuals have yielded a five-factor structure that reflects the theoretically assumed domain structure of MBI. However, a relatively large number of items had rather low factor loadings [22]. Some studies on the factor structure of the Chinese versions of the MBI-C showed cultural influences when answering the questionnaire [23, 24]. In further validation studies on the MBI-C, it showed good internal consistency [24, 25] and test-retest reliability [24–26] as well as satisfactory discriminant [25] and convergent validity. For example, it showed satisfactory convergent validity with the Neuropsychiatric Inventory Questionnaire (NPI-Q; [27]), which is designed to measure NPS in people with dementia [24–26, 28–30]). Furthermore, the MBI-C total score was reported to be a significant predictor of an MBI diagnosis on the basis of the ISTAART-AA criteria [29, 30]. However, the correlations between self-reports and proxy reports appeared to be rather low [22, 26], and there is still no sufficiently validated and consistently applied cut-off value for differentiating between people with and without MBI [31]. Taking these exceptions into account, research results indicate that the MBI-C is a reasonably reliable and valid instrument for assessing persistent NPS. However, regarding the current state of research, this assumption cannot simply be applied to the German self- and proxy-assessed versions of the MBI-C.

In a first validation study [32], the German MBI-C was applied to dyads in interviews and showed an internal consistency in the questionable range (Cronbach’s alpha = 0.64) and only a moderate level of test-retest reliability (ICC = 0.53) for the self-report version. For the self-report version in particular, only some of the assumed correlations with other instruments for assessing construct validity were found and none with the (proxy-rated) Neuropsychiatric Inventory [32, 33]. Finally, the very low mean scores [32] and the fact that almost a quarter of the self-rating participants did not even answer any of the 34 items on the MBI-C indicate that the use of such a detailed instrument has limited benefits for people who do not have severe cognitive impairments and live in private households. In addition to these psychometric limitations, there are other characteristics of the MBI-C that indicate potential opportunities for adjustments. The MBI-C contains several broad items in which different behaviors or characteristics are covered simultaneously (e.g., “Has the person become agitated, aggressive, irritable, or temperamental?“) or in which two questions are included in one item (e.g., “Has the person developed sadness, or does the person appear to be in low spirits? Does he/she have episodes of tearfulness?“). This complexity places greater cognitive demands on the rater and makes the questions appear less comprehensible, an issue that was also criticized by some participants [32]. It should also be noted that in both studies on the German MBI-C [21, 32], as well as in other studies [24, 26, 28–30], the questions were presented in an interview, which allowed participants to ask questions in the event of such uncertainties. Although it would be more economical to record NPS with a questionnaire, particularly precise and comprehensible questions would be needed, as the aforementioned ambiguities in the MBI-C items could potentially lead to items being left unanswered. Some questions concern socially very unacceptable and impaired behavior, assessing, for example, hallucinations, sexually disinhibited behavior, vulgar behavior, or impulsive behavior, such as stealing and gambling. In the study by Riedel-Heller et al. [32], these behaviors were mostly not reported either in the self-report or in the proxy report and had the lowest mean scores in the study by Dibbern et al. [21], which made them seem to be of little relevance in non-demented populations, potentially jeopardizing the acceptance of the instrument. Dibbern et al. [21] reported a completion time for the MBI-C between 5 and 40 min (*M* = 16 min) in non-demented, hospitalized elderly patients. Particularly older people with cognitive impairments may take longer to answer the sometimes demanding questions on the MBI-C, thus posing an additional burden and possibly reducing acceptance of the instrument. In addition to the complexity of some questions, the frame of reference of six months for the assessment of symptoms and the response format also pose considerable cognitive challenges for raters. Furthermore, the MBI-C is suitable only to a limited extent for use in follow-up measurements. The current behavior needs to be compared with previous behavior, and the intensity of the change is rated, not that of the symptom, so further worsening of a symptom at follow-up cannot be recorded if the intensity of the change is perceived as unchanged. As in MCI, the severity of MBI can change, which could be assessed more reliably by measuring the intensity of the symptoms.

Although initial studies have shown that the MBI-C’s psychometric parameters are “sufficient” for the assessment of newly emerged, persistent NPS in later life, its characteristics have pointed out that there is room for optimization. In addition to the MBI-C, there is a need for a supplementary, abbreviated instrument featuring well-accepted, precise, and easily comprehensible items. Such an instrument should also, in principle, be suitable for measuring spontaneous or intervention-caused short-term changes of MBI and, as a short instrument, should require little processing time. In summary, it should be designed to measure MBI as a short-term modifiable state criterion. The aim of the present study was to develop such an instrument, the Mild Behavioral Impairment Scale (MBI-S), as well as to validate it and to investigate its psychometric properties.

## Methods

### Design

Participants were recruited for this cross-sectional validation study in two ways. The survey documents were mailed to informal caregivers living in Bavaria who had participated in the “Benefits of Being a Caregiver” study (recruitment in 2019–2020) and had given their consent to be contacted for future voluntary surveys. In addition, survey documents were distributed to patients in two outpatient medical facilities in Heiligenstadt and Eckental (Bavaria, Germany). Inclusion criteria were being at least 18 years of age, being able to complete a questionnaire in German, and not having dementia. In contrast to the previous study participants who were re-contacted, the individuals recruited in the medical context remained anonymous to the research team. By returning the completed questionnaire, the participants consented to the anonymized use of the information they provided. Data collection began in September 2022 and was completed in January 2023. Approval for the study was obtained from the ethics committee of the Medical Faculty of the Friedrich-Alexander-Universität Erlangen-Nürnberg (No.: 220_20 B).

### Sample

**Fig 1:**
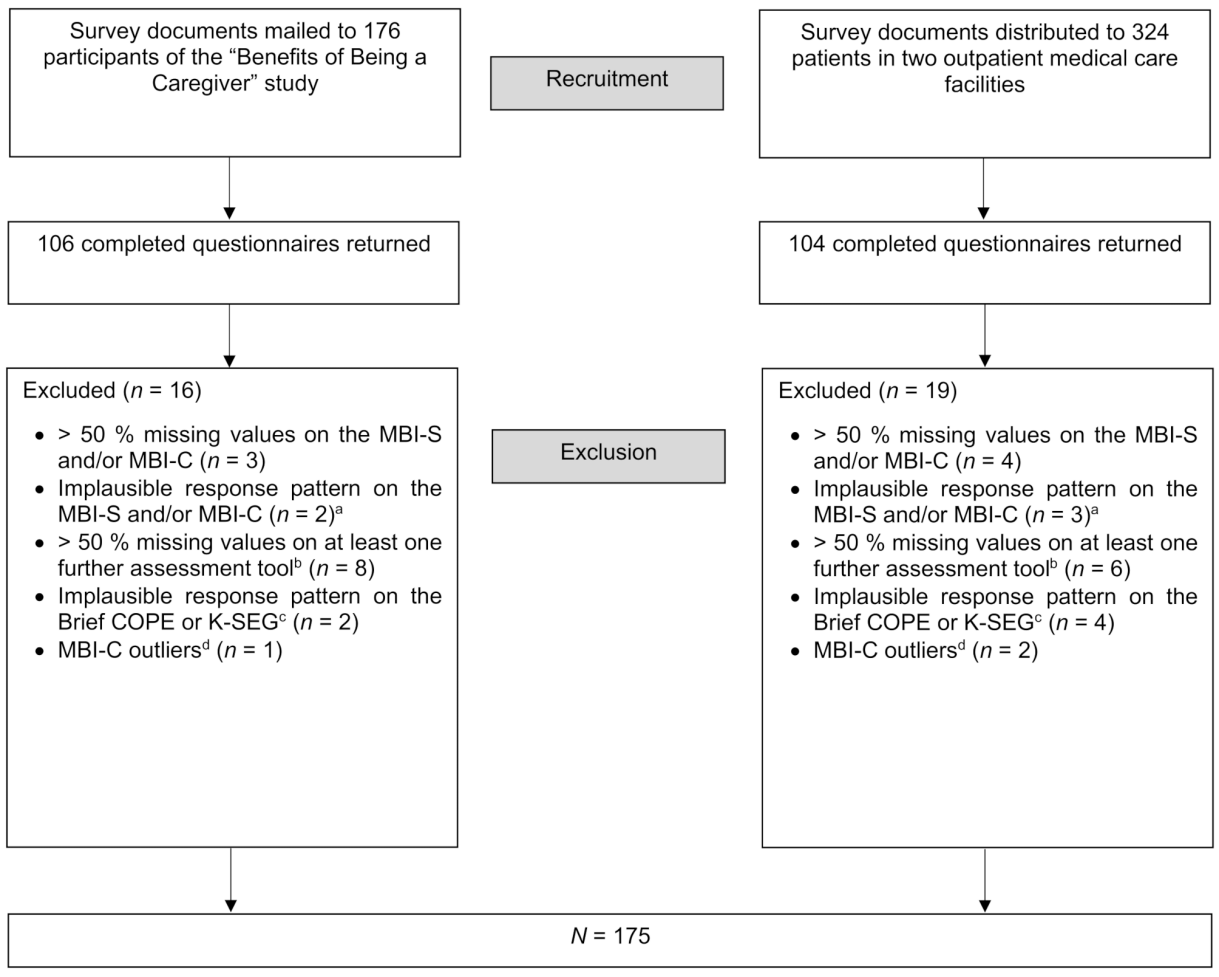
Flow chart of the sampling process. *Notes*. MBI-S: Mild Behavioral Impairment Scale; MBI-C: Mild Behavioral Impairment Checklist; K-SEG: Social Desirability-Gamma Short Scale ^a^ Implausible response pattern refers to > 50% of answers not being interpretable because the application of the two-step response format was not consistent with the given instructions ^b^ Items on sociodemographic characteristics were not considered ^c^ Implausible response pattern refers to all items with a score of 0 ^d^ Extreme outliers were defined as values that fell outside the range of three times the interquartile range from the first quartile down and the third quartile up. These cases were excluded due to a high probability of psychiatric issues that were not statistically controlled for and might bias the results

After 35 cases were excluded (see Fig. 1 for details), the final sample consisted of 175 individuals, 90 of whom were former participants of the “Benefits of Being a Caregiver” study and 85 of whom were anonymous respondents. The process that led to the final sample is shown in Fig. 1. The participants’ mean age was 59.6 years (*SD* = 14.5, range = 18–89), and most of the participants were female (68.6%). One person reported non-binary gender. Most participants were married (66.3%) and were co-residing with someone (82.3%). For educational level, 36% of the sample had nine or fewer years of education, 39.4% had 10 years, 11.4% had 12 to 13 years, and 13.1% had more than 13 years. About half of the participants (49.1%) were employed.

### Instruments

#### Development and description of the Mild Behavioral Impairment Scale (MBI-S)

The adapted version of the German MBI-C [32] served as the basis for developing a shortened instrument, as it is available in a self-report version. In a first step, a focus group consisting of a medical doctor, two psychologists, one of whom was also a psychological psychotherapist, and a doctoral student at the Center for Health Services Research in Medicine at the Uniklinikum Erlangen selected items from the 34-item version of the MBI-C in a structured, predefined, 4-step procedure. As an established, valid, and reliable instrument for assessing NPS in neurodegenerative diseases, the Neuropsychiatric Inventory, especially its questionnaire version, the NPI-Q [27, 33], served as the basis for item selection (Step 1). The NPI-Q assesses 12 symptoms (delusions, hallucinations, agitation/aggression, depression/dysphoria, anxiety, elation/euphoria, apathy/indifference, disinhibition, irritability/lability, motor disturbance, nighttime behaviors, appetite/eating), and the aim was to select MBI-C items (one each) that best represented these symptoms with the greatest possible agreement on content. All participants of the focus group made proposals about which items to select, and if there was consensus that one item best represented a symptom of the NPI-Q, it was selected. Otherwise, the decision was discussed further until consensus was achieved. This process resulted in 10 preliminary items. For the symptoms elation/euphoria and nighttime behaviors, no suitable MBI-C items were available, so the original questions were taken from the NPI-Q and adapted for self-report by the focus group (Step 2). The existing 12 items were revised (Step 3). The process included the adaptation of questions for assessing the current status instead of changes from previous behavior, for example, the item “Do you get up more often at night or sleep more during the day than before?” was rephrased as “Do you get up frequently at night and/or do you sleep a lot during the day?” (Step 3, part 1). In order to avoid ambiguous answers when all the specific symptoms in a question applied versus only some, the symptoms within an item were linked with the wording “and/or.” Items were analyzed and shortened if necessary to reduce ambiguity when a question contained many specific symptoms (Step 3, part 2). For example, “Do you feel very tense, having developed an inability to relax, or shakiness, or symptoms of panic?” was rephrased as, “Do you feel tense in the sense that you are no longer able to relax properly?” Some items were toned down to increase acceptability, for example, the item “Do you believe you are in danger or that others are planning to hurt or steal from you?” was rephrased as “Does it sometimes seem to you that something is going on even though it can’t really be?” (Step 3, part 3). For the MBI-S, the two-step response format of the MBI-C was retained and adapted to the NPI-Q response format and observation period (Step 4). The first step assesses whether a symptom is present, and if it is, a three-level severity score (1 = mild, 2 = moderate, 3 = severe) is additionally assigned, and respondents are asked to indicate the severity of each symptom. In accordance with the NPI-Q, symptoms must have been present in the last four weeks. This time period is shorter than the period of six months used in the MBI-C but can be justified by its better suitability for repeated measurements and higher sensitivity for short-term changes. Furthermore, the MBI-S was not developed to diagnose MBI. The total score ranges from 0 to 36 with higher scores indicating more severe behavioral impairment.

#### Additional measurements

To be able to compare mild behavioral impairment with the original concept, the German version [32] of the **MBI-C** [20] was used. The instrument consists of 34 items that assess five domains: decreased motivation (6 items; e.g., “Have you lost interest in friends, family, or home activities?”), emotional dysregulation (6 items; e.g., “Have you become less able to experience pleasure), impulse dyscontrol (12 items; e.g., “Have you become more impulsive, seeming to act without considering things?”), social inappropriateness (5 items; e.g., “Do you lack the social judgment you previously had about what to say or how to behave in public or private?”), and abnormal perception or thought content (5 items; e.g., “Have you developed beliefs that you are in danger or that others are planning to harm you or steal your belongings?”). Each item is rated for its presence or absence in the last 6 months. The severity of each symptom that was present in the last six months and represents a change from previous persistent behavior is rated on a three-point scale ranging from 0 (mild; significant but without major change) to 3 (severe; very marked or major, dramatic change). The maximum possible overall score on the MBI-C is 102, with a higher score indicating greater behavioral impairment.

Participants’ maladaptive coping behavior was assessed with the German version [34] of the **Brief Coping Orientation to Problems Experienced Inventory** (Brief COPE; [35]). The Brief COPE contains 28 items from 14 scales that are rated on a four-point scale ranging from 0 (not at all) to 3 (very). To assess maladaptive coping, the scores of six of the scales (“venting, “denial,” “substance use,” “behavioral disengagement,” “self-distraction,” “self-blame”) were combined [36]. The total score ranges from 0 to 36 with higher scores indicating that respondents are more likely to use maladaptive coping strategies.

Loneliness was assessed with the German version [37] of the **Three-Item Loneliness Scale** [38]. The instrument comprises three items that are rated on a three-point scale ranging from 0 (rarely) to 2 (often). The total score ranges from 0 to 6, with higher scores indicating greater loneliness.

Social desirability was assessed with the **Social Desirability-Gamma Short Scale** (K-SEG; [39]). The instrument contains three items from each of the two scales “exaggerating positive qualities” (PQ+) and “minimizing negative qualities” (NQ-). Items are answered on a five-point scale ranging from 0 (doesn’t apply at all) to 4 (applies completely). For the analysis, the items of the NQ- subscale were inverse coded. The social desirability scores were computed separately for each subscale by calculating the mean score of the three items of each subscale. The scores ranged from 0 to 4, with higher scores indicating a greater tendency to give socially desirable answers to self-report items.

#### Other measures

Sociodemographic and background characteristics were also assessed. These included, for example, participants’ age and gender, employment, educational level, as well as living situation (co-residing, yes or no). All instruments were administered in German.

### Statistical analysis

#### Description

We calculated the mean, median, and standard deviation for descriptive analyses.

#### Reliability and item analysis

We calculated Cronbach’s alpha as a measure of internal consistency reliability. Cronbach’s alpha was calculated for the overall score. According to Krebs and Menold [40], an alpha of 0.70 or higher is acceptable, and a value of 0.80 or higher is desirable as an indicator that a scale is well-designed. After the item analysis, the difficulty index and discriminatory power were calculated at the item level. While Döring and Bortz [41] recommended an interval from 0.20 to 0.80 for the difficulty index, the discriminatory power was calculated as a deleted item-total correlation. According to Döring and Bortz [41], a discriminatory power of 0.30 to 0.50 can be classified as moderate and a discriminatory power of > 0.50 as high.

All items from the final version of the MBI-S should be important for creating a total score on the basis of psychometric criteria. For this purpose, the internal consistency and the component structure were analyzed. According to a first criterion, items were excluded if Cronbach’s alpha “if item deleted” was higher than the alpha value for the whole scale and their discriminatory power was below 0.30, i.e., they did not differentiate well between individuals with high and low behavioral impairment, that is, they did not capture the target construct well [41].

#### Principal component analysis

To examine the correlation matrix of the MBI-S items, we performed a principal component analysis (PCA). Requirements for PCA were checked with the Kaiser-Meyer-Olkin measure of sampling adequacy, for which a value of at least 0.50 is recommended and a value greater than 0.80 is desirable [42]. In accordance with the Kaiser criterion, only components with eigenvalues > 1 were considered. To determine the number of components to be extracted, a scree plot of the distribution of the eigenvalues was considered, and a parallel analysis [43, 44] was performed. Parallel analysis is used to determine the number of components to retain by comparing the empirical eigenvalues with eigenvalues calculated from randomly generated correlation matrices and retaining only components whose eigenvalues are larger than the corresponding random eigenvalues [44]. In accordance with Backhaus et al. [42], we defined a loading ≥ 0.50 as the criterion for assigning a variable to a component.

According to a second criterion, to ensure the psychometric quality of the MBI-S, items were removed from the scale if they did not have a loading of at least 0.50 on any of the extracted components.

#### Validity

The following hypotheses (H) were tested with regard to convergent (H1 to H3) and discriminant validity (H4):

##### H1

Because the MBI-S and MBI-C [20] were developed to measure the same construct, the two scales were expected to be positively correlated at a high level.

##### H2

Preliminary results indicated a moderate positive correlation between the MBI-C score and the use of negative coping strategies, for example, avoidance [24]. Therefore, a positive correlation between the MBI-S and maladaptive coping captured by the Brief COPE was expected.

##### H3

Meta-analytic results indicated that loneliness is moderately correlated with depression [45] and psychotic symptoms [46]. It also has small to moderate relationships with sleep problems both cross-sectionally and longitudinally [47, 48]. Similarly, strong correlations between loneliness and anxiety have been reported in older individuals [49], further supporting the hypothesis of a relationship between loneliness and NPS. Therefore, a positive correlation was expected between the MBI-S and the Three-Item Loneliness Scale.

##### H4

Previous studies have reported that the number of years of education was only unimportantly and non-significantly related to the MBI-C score [29]. This finding indicates that MBI is independent of the respondent’s educational level. Therefore, we expected no relationship between the MBI-S score and educational level.

To test H1, H2, and H3, the correlations between the MBI-S score and the metric variables were computed as Pearson correlation coefficients (*r*). To test H4, Spearman’s non-parametric rank correlation coefficient (*r*_*s*_) was calculated. According to Döring and Bortz [41], correlations greater than 0.50 are considered high, those between 0.30 and 0.50 are moderate, and those between 0.10 and 0.30 are low. Correlations of less than 0.10 indicate that there is no relevant association. To control for the accumulation of the alpha error from multiple testing, we applied the Benjamini-Hochberg correction [50].

The correlation of the MBI-S score with the sex of the respondents was examined by calculating the point-biserial correlation coefficient (*r*_*pb*_). As self-ratings have often been shown to be confounded with the social desirability response bias [51], the relationship between the MBI-S and the K-SEG was assessed. Therefore, the MBI-S score’s correlations with the PQ + and NQ- subscales of the K-SEG were calculated as Pearson correlation coefficients.

IBM SPSS version 28 for Windows was used for all statistical analyses. The alpha level was set to 5%.

## Results

### The MBI scores and other scales

The mean of the MBI-S score was 5.97 (*SD* = 4.92) with a median of 5.00 (Table 1). The observed scores ranged from 0 to 24. Values in the possible range from 25 to 36 did not occur. For the MBI-C, the mean score was 8.23 points (*SD* = 8.88) with a median of 5.00. There was a greater distribution of low scores. The range of the observed values was 0 to 34 points. Values from 35 to 102 points did not occur.

**Table 1:** Distributions of the scale values

Variable (potential range)	M	SD	Median	Range	Cronbach‘s alpha
MBI-S (0–36)	5.97	4.92	5.00	0–24	0.74
MBI-C (0-102)	8.23	8.88	5.00	0–34	0.89
Loneliness scale (0–6)	1.41	1.69	1.00	0–6	0.83
Brief COPE M (0–36)	8.79	3.58	9.00	1–20	0.47
K-SEG PQ+ (0–4)	2.84	0.64	3.00	1.00–4.00	0.61
K-SEG NQ- (0–4)	3.60	0.52	3.67	0.67–4.00	0.61

Note. MBI-S: Mild Behavioral Impairment Scale; MBI-C: Mild Behavioral Impairment Checklist; Loneliness scale: Three-Item Loneliness Scale; Brief COPE M: Subscale maladaptive Coping of the Brief COPE; K-SEG PQ+: Subscale exaggerating positive qualities of the Social Desirability-Gamma Short Scale; K-SEG NQ-: Subscale minimizing negative qualities of the Social Desirability-Gamma Short Scale

### Reliabilities and item analyses

Cronbach’s alpha was 0.743 for the 12-item scale. Cronbach’s alpha “if item deleted” was below the value for the entire scale for 10 of the 12 items (see Table 2). Only items 6 and 11 had a Cronbach’s alpha “if item deleted” above the value for the entire scale. The discriminatory power of the items ranged from − 0.09 to 0.60 (Table 2). The discriminatory power of items 3, 4, 5, and 7 was high (> 0.50), while four items (items 6, 8, 11, and 12) had low discriminatory power (< 0.30). Item difficulties ranging from 0.03 to 0.27 turned out to be low overall, with five items (items 2, 4, 5, 7, and 9) falling within the target range of 0.20 to 0.80. The remaining seven items (items 1, 3, 6, 8, 10, 11, and 12) had values below 0.20.

Applying the first criterion to ensure the psychometric quality of the MBI-S score resulted in the exclusion of items 6 and 11. They were excluded because the removal of each item resulted in an increase in Cronbach’s alpha for the scale to 0.766 (item 6) and 0.749 (item 11), and their discriminatory powers of − 0.09 and 0.18 were low. Thus, items 6 and 11 were not considered further in subsequent analyses.

**Table 2:** Characteristics of the items of the MBI-S

Item	M	SD	Discriminatory power	Item difficulty	Cronbach’s alpha “if item deleted“^a^
1) Do you get up frequently at night and/or do you sleep a lot during the day?	0.55	0.88	0.38	0.18	0.726
2) Has your body weight changed (because you unintentionally eat more or less)?	0.76	0.95	0.37	0.25	0.728
3) Do you feel agitated and/or even aggressive?	0.44	0.72	0.55	0.15	0.707
4) Do you feel sad and/or down?	0.73	0.94	0.56	0.24	0.699
5) Do you feel tense in the sense that you are no longer able to relax properly?	0.81	0.96	0.52	0.27	0.706
6) Do you feel remarkably well in the sense that you are cheerful for no real reason?^b^	0.23	0.57	− 0.09	0.08	0.766
7) Do you have less drive to deal with your obligations and/or interests?	0.66	0.95	0.60	0.22	0.692
8) Are you often impulsive (e.g., do things without thinking beforehand)?^b^	0.23	0.56	0.26	0.08	0.737
9) Do you easily become impatient? (e.g., do you have problems dealing with delays and/or waiting for something? )	0.69	0.92	0.42	0.23	0.720
10) Are there actions that you repeat over and over again in the same way due to inner pressure?	0.36	0.76	0.45	0.12	0.717
11) Does it sometimes seem to you that something is going on even though it can’t really be?^b^	0.45	0.81	0.18	0.15	0.749
12) Do you sometimes have the impression that you can hear people speaking even when no one is present?^b^	0.08	0.35	0.24	0.03	0.741

Note. ^a^ Cronbach’s alpha (12 Items) = 0.743; ^b^ Item not included in the final version of the Mild Behavioral Impairment Scale

### Principal component analysis

**Fig 2:**
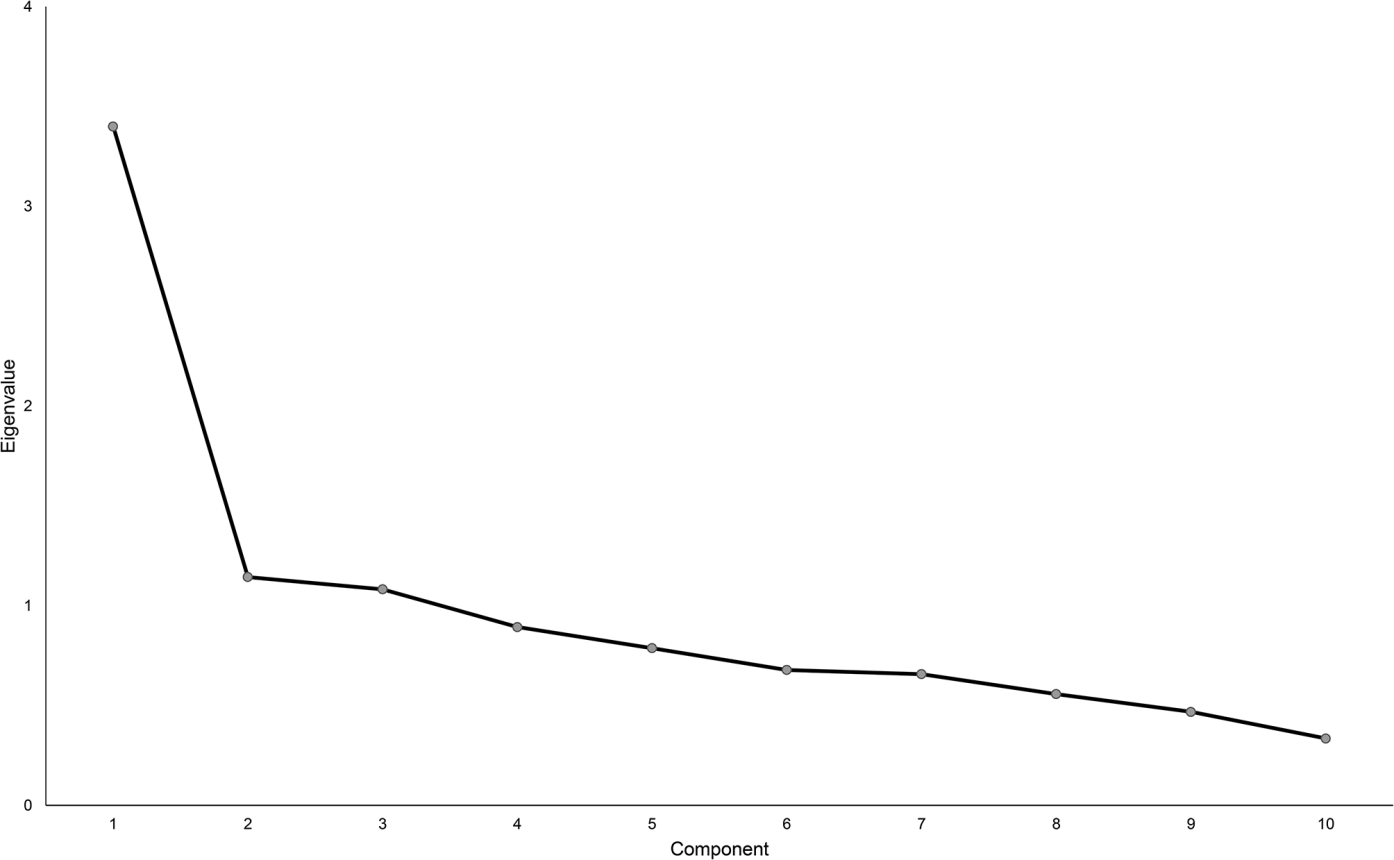
Scree plot for the PCA of the MBI-S (with the remaining 10 items)

A PCA was performed with the 10 remaining items (items 1, 2, 3, 4, 5, 7, 8, 9, 10, 12). The Kaiser-Meyer-Olkin parameter was 0.81, indicating sampling adequacy. The correlations between items ranged from 0.04 to 0.61 with a median of 0.25. The PCA revealed three components with eigenvalues greater than 1.0, but the first component’s eigenvalue was about three times larger than the other two (3.40 vs. 1.14 vs. 1.08). The first component therefore explained three times the amount of variance (34.0% vs. 11.4% vs. 10.8%). The graphical analysis of the scree plot justified the one-component solution (see Fig. 2), and the results of the parallel analysis provided further empirical justification for retaining one component. A one-component solution meant that a total score could be calculated for the MBI-S. When one component was extracted, the loadings ranged from 0.28 to 0.76 (see Table 3). Seven items (items 1, 3, 4, 5, 7, 9, 10) had component loadings greater than 0.50. Item 2 had a loading of 0.48, deviating from 0.50 by only 0.02. By contrast, items 8 and 12 showed loadings that were significantly smaller than 0.50 (i.e., 0.28 and 0.31, respectively).

**Table 3:** Loadings of the remaining 10 items of the MBI-S

Item	Loading
1) Do you get up frequently at night and/or do you sleep a lot during the day?	**0.54**
2) Has your body weight changed (because you unintentionally eat more or eat less)?	0.48
3) Do you feel agitated and/or even aggressive?	**0.70**
4) Do you feel sad and/or down?	**0.74**
5) Do you feel tense in the sense that you are no longer able to relax properly?	**0.70**
7) Do you have less drive to deal with your obligations and/or interests?	**0.76**
8) Are you often impulsive (e.g., do things without thinking beforehand)?	0.31
9) Do you easily become impatient? (e.g., do you have problems dealing with delays and/or waiting for something? )	**0.55**
10) Are there actions that you repeat over and over again in the same way due to inner pressure?	**0.57**
12) Do you sometimes have the impression that you can hear people speaking even when no one is present?	0.28

Note. Loadings ≥ 0.50 are printed in bold

Applying the second criterion for ensuring the psychometric quality of the MBI-S (i.e., items that did not have a component loading equal to or greater than 0.50 were excluded) resulted in the exclusion of items 8 and 12. Their loadings were substantially below the defined limit, and they therefore could not be assigned to the component. Items 8 and 12 additionally had low correlations with the other items (all *r* between 0.04 and 0.27) as well as discriminatory power in the insufficient range (< 0.30). Item 2 also met the second criterion but deviated only slightly (0.02) from the defined cut off and had no additional psychometric issues, so we could justify retaining this item. The final version of the MBI-S thus consisted of eight items (see Additional file 1 for the complete questionnaire).

### Characteristics of the final eight-item version of the MBI-S

The mean of the final MBI-S score was 4.99 points (*SD* = 4.50) with a median of 4.00. The distribution of the scores covered the range from 0 to 22. Possible values of 23 and 24 did not occur. Cronbach’s alpha increased from 0.74 for the original 12-item version to 0.79 for the final eight-item version.

### Convergent and discriminative validity

We adjusted the *p*-values by applying the Benjamini-Hochberg correction [50, 52]. There was a strong correlation between the score from the final eight-item MBI-S and the MBI-C score (*r* = 0.80, *p* = 0.002). Furthermore, the MBI-S score was moderately correlated with the maladaptive coping scale score from the Brief COPE (*r* = 0.32, *p* = 0.002) and strongly correlated with the score from the Three-item Loneliness Scale (*r* = 0.52, *p* = 0.002). There was no statistically significant association between the MBI-S and level of education (*r*_*s*_ = − 0.06, *p* = 0.504). All three hypotheses on convergent validity were supported, that is, the MBI-S was convergent with the MBI-C, the extent of maladaptive coping, and loneliness. Hypothesis 4 on discriminant validity was supported, too, meaning that the MBI-S score was independent of educational level.

Beyond these findings, there was a low but significant correlation between the MBI-S and the sex of the participants in the direction of higher scores for females (*r*_*pb*_ = − 0.18, *p* = 0.015). The mean score was 3.76 (*SD* = 3.66) for males and 5.53 (*SD* = 4.75) for females. Only males and females were considered for this analysis, as the non-binary gender group was not sufficiently represented in the sample (*n* = 1). The correlation of the MBI-S score with the PQ + subscale from the K-SEG was − 0.29 (*p* <.001), and the correlation with the NQ- subscale was − 0.07 (*p* = 0.383). There was a small but significant correlation with PQ + in the sense that positive qualities were less exaggerated in participants with higher MBI-S scores. By contrast, there was no relationship between the MBI-S and NQ-; that is, there was no relationship with the tendency to understate negative qualities.

## Discussion

The aim of this study was to develop and validate a brief instrument for the time-efficient assessment of self-reported NPS in non-demented individuals. The multi-step development of the MBI-S began by selecting the items from the MBI-C deemed best for assessing the NPS sub-domains that were included in the NPI-Q [27]. After the items were selected, they were adapted, and the resulting 12-item questionnaire was subsequently distributed to a sample of family caregivers and people recruited from an outpatient medical context to validate the instrument. In the item analysis, the items for measuring elation/euphoria (item 6) and delusions (item 11) did not meet the psychometric quality requirements and were removed. A subsequent PCA of the 10 remaining items showed that the loadings of the disinhibition (item 8) and hallucination (item 12) items on the extracted component clearly deviated from the defined limit (≥ 0.50) for assignment to a component, and these two items were thereby removed. The final MBI-S consists of eight items and measures MBI as a short-term modifiable state criterion.

### Validity of the mild behavioral impairment scale

To ensure content validity, the MBI-S items were selected to cover the 12 NPS domains from the validated NPI-Q [27]. The number of items in the first version of the MBI-S corresponded to that of the NPI-Q, as did the response format, the scaling of the responses, as well as the assessment period of four weeks. As the MBI-S is intended to assess NPS in the pre-dementia stage, most of the specific items representing the NPS domains (7 of 8 items in the final version) stem from the MBI-C. However, due to the modality of item selection and the empirically driven item reduction, only the MBI domains of reduced motivation, affective dysregulation, and impulse dyscontrol [11] are represented on the MBI-S, whereas no items could be assigned to the domains of social inappropriateness or abnormalities of perception or thought content, thus impacting the content validity of the MBI-S. However, the MBI-S was not developed as a diagnostic tool for MBI, which would require coverage of all domains according to the ISTAART-AA criteria. Instead, it was developed for the brief assessment of NPS in non-demented individuals [11]. In addition, the items from these domains had the lowest prevalence in previous research on the German MBI-C [21, 32]. This finding indicates that these domains are not as relevant as the others. Therefore, the MBI-S can be described as a valid instrument in terms of content.

All eight remaining MBI-S items demonstrated sufficient psychometric quality. The discriminatory power ranged from 0.37 to 0.60, and all eight items contributed to the internal consistency reliability of the scale The appropriateness of the scale construction was reflected in an acceptable internal consistency (Cronbach’s alpha = 0.79) for the eight-item MBI-S. The item difficulty indices were in the range of 0.12 to 0.27 and were thus rather low, which showed that the participants experienced relatively few behavioral impairments. This result is in line with previous findings on the German MBI-C in a community sample [32]. None of the inter-item correlations exceeded 0.61, indicating that the scale contains no redundant items. Furthermore, the one-component solution from the PCA was an appropriate result that was based on eigenvalues, the scree plot, and a parallel analysis. Thus, the calculation of a total score for the MBI-S was also empirically justified. The eight items that remained on the final version had loadings greater than 0.48, thus providing further justification for the single-component solution. The items on disinhibition (item 8) and delusions (item 11), which were not assigned to the component due to their low loadings (≤ 0.31) could potentially form a separate component that is characterized by stronger clinical impairment. With only eight items, the time required to complete the scale is also quite short. The MBI-S is therefore an economical instrument.

All three hypotheses on the convergent validity of the MBI-S were supported. There was a significant strong positive correlation between the MBI-S score and the MBI-C total score (H1), which are intended to measure the same construct. Thus, the construct validity of the MBI-S was supported by a demonstration of convergent validity. There was also a significant moderate positive correlation between the MBI-S and the maladaptive coping scale from the Brief COPE (H2), which is consistent with the finding of a moderate correlation between the MBI-C total score and negative coping strategies by Lin et al. [24]. It has been suggested that NPS in dementia may result from an imbalance between stressors and effective coping opportunities [53], and a small qualitative study also showed evidence of this mechanism in people with MCI through an intensification of NPS from the use of negative coping strategies [54]. This mechanism could explain the correlation found in the current sample. The association of NPS with maladaptive coping in non-demented individuals requires further research, as the reduction of dysfunctional coping strategies could represent a potential intervention for NPS [54]. In line with previous findings on relationships between certain NPS and loneliness, we found a significant strong positive correlation between the MBI-S and Three-Item Loneliness Scale (H3). NPS can be associated with a reduced level of social functioning [55, 56], which in turn is related to greater loneliness [57], for example, due to interpersonal problems. As loneliness represents a risk factor for mental and physical health and well-being [58], healthcare professionals should be educated about possible comorbid loneliness in people with NPS to initiate interventions. The hypothesis on discriminant validity (H4) was also supported. As assumed, the MBI-S score had a quasi-zero correlation with participants’ educational level, and thus, the MBI-S score is independent of education. This result is in line with previous empirical results [29].

Concerning social desirability, it is noticeable that the NQ- scale from the K-SEG [39] was not associated with the MBI-S score (*r* = −0.07). This result demonstrates independence of the MBI-S score from the tendency to minimize negative qualities. Concerning the PQ + scale, a moderate correlation (*r* = − 0.29) in the direction of less exaggeration of positive qualities with a higher MBI-S score was found. Thus, the relative robustness of the MBI-S against socially desirable response behavior could be demonstrated.

The MBI-S score was weakly related to gender, with a slightly higher average score for females (1.8 points). Previous findings on gender differences in MBI have been inconclusive, but the cognitive status of participants was identified as a potential moderating variable [59]. As we did not assess cognitive performance in our study, its influence on gender differences in MBI could not be explored One explanation for the gender difference we found could be the selection of the MBI-S items. Half of the items (items 1, 2, 4, 7) measure symptoms that also represent diagnostic characteristics of depression (depressed mood, reduced drive, sleep disturbances of all kinds, changes in appetite; [60]). Depressive disorders in turn appear to be more prevalent in females [61]. This possible relationship between depression and the MBI-S score should be considered in future research, too.

### Limitations

The study participants represented a convenience sample recruited in clinical and non-clinical settings. Therefore, the results cannot be generalized to a well-defined population. We did not have any proxy ratings that could be compared with the MBI-S self-ratings. Consequently, the results should be interpreted in light of potential biases inherent to such measurements. Also, it was not possible to measure cognitive parameters in the sample. Therefore, we could not analyze the associations of the MBI-S with MCI or non-MCI. In light of these considerations, we advise that the MBI-S be utilized in clinical contexts as a supplementary tool. To achieve a thorough evaluation of the construct, it is recommended to complement the MBI-S with additional, more objective measures, including direct cognitive assessments or behavioral observations. Psychiatric disorders were not controlled for, so individuals may have reported symptoms of unknown underlying diseases in the MBI-S and MBI-C. This problem was counteracted by excluding individuals with extremely high MBI-S and MBI-C scores from the sample. Furthermore, as the MBI-S assesses current NPS within the last four weeks, a one-time application of the instrument is not suitable for assessing MBI with respect to its clinical definition, which requires a persistence of symptoms of at least six months [11]. Finally, the reported results were based on cross-sectional analyses, so no conclusions can be drawn about the MBI-S’s sensitivity to change.

## Conclusion

The construction of the MBI-S is theoretically based, first, on the NPS sub-domains referred to in the NPI-Q [27], and second, on the extraction and adaption of appropriate MBI-C items. It resulted in a short, self-rated questionnaire with eight items with a total score that is based on one empirical component. The MBI-S score is convergent with loneliness, maladaptive coping, and the MBI-C total score. Additionally, we found that the MBI-S score is largely robust to social desirability and is independent of education. These findings are important for a broad application of the instrument. Future research is necessary to strengthen insights into the MBI-S by using other clinical and non-clinical study groups to gain knowledge about other – especially clinical – parameters of MBI and by conducting longitudinal studies to examine the extent to which the MBI-S is sensitive to change.

## Electronic supplementary material

Below is the link to the electronic supplementary material.

Supplementary Material 1

## Data Availability

The data sets used and/or analyzed during the current study are available from the corresponding author upon reasonable request.

## Abbreviations

ICC: Intraclass Correlation Coefficient
ISTAART-AA: International Society to Advance Alzheimer’s Research and Treatment– Alzheimer’s Association
K-SEG: Social Desirability-Gamma Short Scale
MBI-C: Mild Behavioral Impairment Checklist
MBI-S: Mild Behavioral Impairment Scale
MBI: Mild Behavioral Impairment
MCI: Mild Cognitive Impairment
NPI-Q: Neuropsychiatric Inventory Questionnaire
NPS: Neuropsychiatric Symptoms
NQ-: “Minimizing negative qualities” scale from the K-SEG
PCA: Principal component analysis
PQ+: “Exaggerating positive qualities” scale from the K-SEG
*r*: Pearson correlation coefficient
*r*_*pb*_: Point-biserial correlation coefficient
*r*_*s*_: Spearman’s non-parametric rank correlation coefficient
