# Supplementary material for: Validation of the Mild Behavioral Impairment Scale (MBI-S) for brief self-assessment of Mild Behavioral Impairment in people without dementia
Source: Ann Gen Psychiatry. 2025 May 29;24:35. doi: 10.1186/s12991-025-00566-w (PMC12123818; doi:10.1186/s12991-025-00566-w)
Supplement: Supplementary file 1 — Supplementary Material 1 [file 12991_2025_566_MOESM1_ESM.docx]

**Additional File 1** Mild Behavioral Impairment Scale – English version

| The following questions focus on your feelings and behavior over the  last four weeks.  Answer "no" if the statement does not apply to you.  Answer "yes" if the statement applies to you and indicate how severely you are affected (mildly, moderately, or severely). | | | | | | | |
| --- | --- | --- | --- | --- | --- | --- | --- |
|  |  |  |  |  | |  | |
|  |  | | If Yes : Severity? | | | | |
|  | Yes | No | Mild | | Moderate | | Severe |
| Do you get up frequently at night and/or do you sleep a lot during the day? | **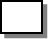** | 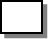 | **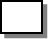** | | **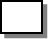** | | **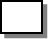** |
| Has your body weight changed (because you unintentionally eat more or less)? | **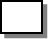** | **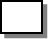** | **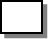** | | **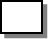** | | **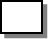** |
| Do you feel agitated and/or even aggressive? | 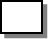 | **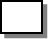** | 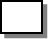 | | 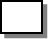 | | 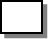 |
| Do you feel sad and/or down? | 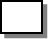 | **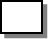** | 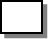 | | 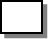 | | 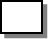 |
| Do you feel tense in the sense that you are no longer able to relax properly? | 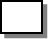 | **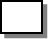** | 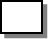 | | 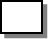 | | 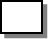 |
| Do you have less drive to deal with your obligations and/or interests? | 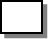 | **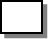** | 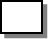 | | 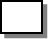 | | 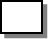 |
| Do you easily become impatient? (e.g., do you have problems dealing with delays and/or waiting for something?) | 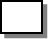 | **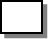** | 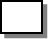 | | 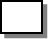 | | 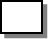 |
| Are there actions that you repeat over and over again in the same way due to inner pressure? | 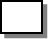 | **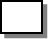** | 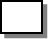 | | 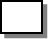 | | 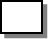 |
